# Neuropsychiatric Symptoms after Liver Transplantation in a 65-Year-Old Male Patient

**DOI:** 10.3390/brainsci12121721

**Published:** 2022-12-16

**Authors:** Cesar Bugallo-Carrera, David Facal, Cristina Domínguez-Lenogue, Vanessa Álvarez-Vidal, Manuel Gandoy-Crego, José Caamaño-Ponte

**Affiliations:** 1 Asociación de Familiares de Enfermos de Alzheimer de Fisterra e Soneira—Afafes. Cee, 15270 A Coruña, Spain; 2 Department of Developmental Psychology, University of Santiago de Compostela, 15782 Santiago de Compostela, Spain; 3 Hospital de Día de Procesos, Servicio de Geriatría, Hospital Universitario Lucus Augusti, 27003 Lugo, Spain; 4 Department of Psychiatry, Radiology, Public Health, Nursing and Medicine, University of Santiago de Compostela, 15782 Santiago de Compostela, Spain; 5 Medical Department, Sanitas Hospitals, 15004 A Coruña, Spain

**Keywords:** liver transplant, immunosuppression, neurotoxicity, psychosis, depression, anxiety, cognitive intervention

## Abstract

The development of immunosuppressants has been key for the advancement of solid organ transplant surgery. Specifically, cyclosporine, tacrolimus, or everolimus have significantly increased the survival rate of patients by reducing the risk of a rejection of the transplanted organ and limiting graft-versus-host disease. We report the case of a 65-year-old man who, after undergoing a liver transplantation and receiving an immunosuppressive treatment with cyclosporine and everolimus, presented severe obsessive, psychotic, and behavioral symptoms over the past three years, and describe the pharmacological and non-pharmacological interventions implemented against these symptoms. In this case, the immunosuppressants used have been cyclosporine and, preferably, everolimus. On the other hand, potential adverse reactions to the treatment have been observed, including neuropsychiatric symptoms such as tremor, anxiety, dysthymia, psychosis, and behavioral disorders, which make it necessary to use corrective psychoactive drugs such as benzodiazepines, antidepressants, and antipsychotics, combined with non-pharmacological interventions. A transversal approach, from the medical and psychosocial disciplines, facilitates success in managing neuropsychiatric symptoms after soft organ transplants.

## 1. Introduction

The first-choice therapeutic alternative for solid organ failure—heart, lungs, pancreas, or liver—is transplantation. This type of intervention requires an interdisciplinary approach to comply with the criteria of efficiency and control possible clinical, psychosocial, and ethical complications. In the case of allograft transplants, the possibility of rejection increases since the immune system of the recipient recognizes the graft as foreign with the consequent response and clinical difficulties. To avoid graft rejection and to guarantee the success of the transplant, immunosuppressive therapy is key; however, limiting the responses of the immune system may contribute to the appearance of organic and psychological complications. In addition, a treatment with immunosuppressants may be extended for longer periods and the risk of neurotoxicity is high [1,2]. Additionally, the postoperative effect of anesthetics can also contribute to neuropsychiatric symptoms and this effect could be exacerbated by additional immunosuppressants.

Regardless of the organ to be transplanted, the most commonly used immunosuppressants are corticosteroids, calcineurin inhibitors (cyclosporine and tacrolimus), purine metabolism inhibitors (azathioprine and mycophenolate), rapamycins (sirolimus and everolimus), immunosuppressive immunoglobulins, and monoclonal/polyclonal antibodies [3]. Transplant complications range from graft rejection to infections, neoplasms, and toxic effects in different organs [4,5].

Calcineurin inhibitors (CIs) (cyclosporine and tacrolimus) selectively inhibit the T lymphocyte activation by blocking their transcription, needed for the production of cytokines. Specifically, CIs inhibit the production of lymphokines, such as Interleukin IL-2 or FKBP, limiting the proliferation of cytotoxic T lymphocytes. Its clearance is carried out by hepatic oxidation through the cytochrome P450 isoenzyme 3A4. Monitoring the drug concentrations in the blood is advisable due to its great pharmacokinetic variability that may lead to dose-dependent adverse reactions as nephrotoxicity is associated with arterial hypertension and neurological symptoms (tremor, paresthesia, or seizures). CIs are metabolized in the liver; thus, interactions may occur with some antibiotics, antifungals, selective serotonin reuptake inhibitors (SSRIs), or calcium antagonists.

Rapamycins inhibit the nuclear protein mTOR by blocking the lymphocyte regulatory kinase, arresting the cell cycle, and inhibiting the lymphocyte response to stimulation with cytokines. Rapamycins bind to the immunophilin FKBP12 and interact with the mTOR protein, blocking the proliferation of T lymphocytes and the synthesis of antibodies. Their elimination depends on cytochrome P450 (CYP3A4 isoenzyme), which may lead to interactions with drugs that induce or inhibit this isoenzyme. Adverse reactions such as nephrotoxicity, metabolic, and hematological alterations, as well as neurotoxicity and psychiatric disorders, occur with Cis and rapamycins [6,7].

There is a close relationship between the immune system and the CNS and the interest on this relationship has intensified since the mid-20th century [8]. The development of psychoneuroimmunology has allowed for identifying different autoimmunity markers such as interleukins, their relationship with T cells, TNF and macrophages, their peripheral and central proinflammatory and anti-inflammatory role, and their involvement with the lymphatic and neurohormonal systems. Consequently, knowledge has grown on different inflammatory biomarkers and a whole series of etiopathogenic hypotheses and therapeutic strategies in pathologies such as autoimmune encephalitis, Alzheimer’s disease, psychosis, anxiety disorders, and affective disorders [9,10,11].

The use of immunosuppressive drugs seems to increase the presence of neuropsychiatric symptoms—potential adverse drug reaction (ADR)—in patients receiving orthotopic liver transplants and other transplants [12,13,14,15]. The most prevalent neurotoxicity-related symptoms are delirium, motor disturbances, mood disorders, nervousness, anxiety, hallucinations, or changes in thought and body image.

Considering that immunosuppressive treatment must be maintained indefinitely to ensure patient survival and the potential comorbidity and pharmacological interactions, mental problems must be addressed through psychosocial intervention and, if necessary, the use of psychotropic drugs. Due to the nature of the symptoms, the most commonly used drugs are antidepressants and antipsychotics. In the case of psychosocial interventions, the therapeutic alliance of clinical and psychosocial professionals with the patient and family or close friends is necessary [16,17].

In this work, we present a case of neuropsychiatric disorder related with the use of immunosuppressive agents and discuss the importance of an interdisciplinary approach to deal with these symptoms.

## 2. Case Report

We report a 65-year-old male patient who is right-handed, a resident in a rural area of A Coruña (Galicia, NW of Spain), married, with two children, and with a primary education. Diagnosed with liver cirrhosis due to an alpha-1 antitrypsin deficiency (heterozygous ZZ), he underwent a transplantation in 2019. Since then, he has presented heterogeneous neuropsychiatric symptoms, highlighting confusion syndrome, delusion of theft, ruin and jealousy, intrusiveness, physical and verbal agitation, insomnia, and mood disorders, with the consequent social and job-related effects, assessed during the study with different items from the Cornell Scale for Depression and the NPI-Q.

### 2.1. Medical History

The patient’s medical record indicates that until 2018, he had bronchial asthma, gallstones (cholelithiasis), and C4-C5 acute infectious spondylitis due to Streptococcus agalactiae complicated with epidural and paravertebral abscesses. There was no record of allergies or hypersensitivity reactions to drugs, a high blood pressure, dyslipidemia, diabetes mellitus, alcoholism, or other toxic habits.

During 1985, while working in Switzerland, he presented a syndrome of depression reactive to the traumatic amputation of three fingers of his left hand after an occupational accident, with the impossibility of returning to work, requiring admission to the acute unit of the psychiatry service. In 2005, he was admitted to a psychiatric hospital for a depressive disorder related to family problems without psychotic symptoms.

In 2018, he was diagnosed with liver cirrhosis due to an alpha-1 antitrypsin deficiency and had an organ transplant in April 2019. During the time interval between the diagnosis of cirrhosis and transplant, the patient was admitted to the hospital several times due to hydropic decompensation and hepatic encephalopathy. During the liver transplantation, a hemodynamic instability occurred secondary to rheumatic valve disease with double mitral and aortic lesions that required a valve replacement with a mechanical prosthesis. Other complications during his stay with the cardiology service were pulmonary thromboembolism, atrial fibrillation, congestive heart failure, and decompensation due to mycophenolate, cytomegalovirus infection, having seizures, repeated confusion syndrome, as well as critical illness polyneuropathy (CIP), all conditions which were successfully controlled.

### 2.2. History of the Disease

The patient was hospitalized in June 2020 in the gastroenterology service for suspected hepatic encephalopathy after two months of highly fluctuating behavioral changes. He was evaluated by the neurology service due to the torpid evolution of his cognitive and behavioral symptoms; a cranial CT scan was performed, as well as routine blood, urine, and cerebrospinal fluid (CSF) tests. No relevant alterations were found. A brain MRI study showed a small area of encephalomalacia in the left middle frontal gyrus, in relation to the cerebral hypoxia secondary to the cardiac dysfunction.

Between September and October 2020, the patient remained hospitalized in the Neurology service due to a behavioral alteration, as stated in the discharge summary.

Different complementary tests were carried out to identify the etiology of the dementia: blood tests (result: mixed anemia), serology (HIV, lues, Borrelia, toxoplasma, Brucella, cytomegalovirus), metals, porphyria, bacteria, tumor (CA 125: 45), and autoimmune markers, with significant findings from the cerebrospinal fluid analysis: proteins 110, later 67, and the rest without alteration. Different neuroimaging tests (cranial CT scans and MRI) suggested probable amyloid angiopathy and mild diffuse cortical atrophy. Progressive electroencephalopathy with the diffuse slowing of the brain bioelectric activity due to frequent theta waves was accompanied by the episodic peak waves of temporal predominance. Liver transplantation post-surgical changes were observed in the chest, abdomen, and pelvis CT scans, without the significant involvement of the transplanted organ. Few small simple renal cysts and prostatic hypertrophy were identified. The chest study revealed minimal pleural effusion and signs of pulmonary hypoventilation.

The diagnostic hypothesis of the episode was “progressive subacute clinical picture of behavioral alteration with psychotic symptoms, of unknown etiology”, “tonic-clonic or convulsive seizures (formerly known as grand mal seizure)”, and “chronic depressive syndrome”.

### 2.3. 2021–2022 Follow Up

The patient follow-up was carried out by the hospital services of gastroenterology, cardiology, neurology, and psychiatry for the control and adjustment of the therapy. Despite the follow-up, some confusion episodes were reproduced, which required hospitalization. In March 2021, the patient maintained behavioral symptoms that limited the therapeutic possibilities, predominantly obsessive ideas, delusions of theft and ruin, Capgras syndrome, and agitation. The scoring for the Mini-Mental State Examination, Barthel Index, and Lawton and Brody index were 30/30, 100/100, and 5/8, respectively, which is expected for a male of his age. Table 1 shows the pharmacological treatments from 2020 to 2022.

The at-home clinical assessment carried out in June 2022 showed an adequate interaction. No signs/symptoms were observed in the general examination (except for the slightly globular abdomen and surgical stigmata) or neurological examination. The heart rate was 82 beats/min, the blood pressure was 110/65 mmHg, the O_2_ saturation was at 97%, the Memory Impairment Screening score was 6/8 (3 out of 4 possible in the 1st attempt) and 8/8 (4 out of 4 in the 2nd attempt), the Goldberg Anxiety and Depression Rating Scale score was 3 (anxiety domain) and 5 (depression domain), and the Neuropsychiatric Inventory (NPI-Q) score was 28.

### 2.4. Psychosocial Intervention

Regardless of what was done through the public health system in Galicia following the established pre- and post-transplant criteria, the patient followed a socio-community therapeutic program in the local Alzheimer Association. He was treated using the STIMULUS platform https://stimuluspro.com (accessed on 4 November 2022) in individual home sessions twice a week between April and May 2021. A cognitive intervention is the treatment of choice in this type of entity, not focusing only on the cognitive domain but also on the affective and relational aspects (e.g., self-esteem through performance, therapeutic relationships with the professional potentially generalizable to other persons involved in the treatment, etc.). The pre–post intervention assessment was conducted by an experienced psychologist with a master’s degree in psychology (author 1). The intervention was conducted by a psychologist with a master’s degree in psychogerontology (author 2). The intervention was conducted in a home environment with the participation of the family.

Computerized cognitive training is recommended in older patients with mild or mild–moderate levels of cognitive impairment as a way of improving the presentation of stimulation materials and maximizing the patient’s motivation. Developed with the aim of offering a useful tool for intervention, Stimulus is a software for cognitive stimulation and rehabilitation offering a playful and attractive support for users. It is based on a series of interactive exercises (+50) that train 10 functional areas (attention, perception, working memory, long-term memory, calculation, reasoning, executive functions, visuomotor functions, language, and speed). The stimulus was conceived as a resource at the service of the socio-sanitary, clinical, or educational professionals, also providing the possibility of being used in the domestic sphere. It has a scientific base, since both in its definition process and its review and validation it had a scientific committee made up of researchers pertaining to the Department of Psychology of the University of Jaen, Spain.

It is worth noting that the intervention occurred during the SARS-CoV-2 pandemic (prolonged confinement, slow lifting of restrictions, and changes in the care systems for the elderly [18]), with the consequent effects on cognitive processes and mental health in frail individuals [19]. Prior to the intervention, an analysis was carried out to establish the cognitive, affective, and behavioral aims, specifically in relation to the delusional activity and agitation. The results of the assessment during the intervention program are shown in Table 2. In the pre-intervention assessment, the scores suggest that both the general cognitive functioning (MoCA) [20] and executive functions (FAB-E) [21] are impaired. Subjective memory complaints were assessed with the MFE according to normative data collected by Delgado et al. [22], depressive symptomatology was measured with the Cornell Scale [23], and delusions, agitation, apathy, disinhibition, and irritability with the NPI-Q [24] and the MBI-C [25]. The training sessions focused on general cognitive activities, emphasizing executive functions and, complementarily, emotional and behavioral aspects, in the context of a broader multidisciplinary intervention.

The analysis of the results during the training period using the STIMULUS platform indicated that the patient improved progressively, with score increases at each session. The mean response time was reduced, the number of exercises in each session and the number of correct answers progressively increased, with a considerable decrease in the errors (Table 3). The incidence of neuropsychiatric symptoms conditioned the efficiency of the intervention.

## 3. Discussion

Many studies have shown a relationship between an organ transplantation, neurotoxicity secondary to the use of immunosuppressive drugs, and neuropsychiatric symptoms. The prevalence of symptoms such as mania, delusions, pseudo-perceptions or depression vary, and all are associated with the neurotoxic processes of different entities. Up to 50% of organ recipients present delirium [26,27] and anxiety–depression symptoms occur in 10–33% of the cases. Craven [28] and Katijiri [29] report the presence of frequent psychotic symptoms, hallucinations, and mania linked with the use of cyclosporine and tacrolimus [30]. Moreover, the use of tacrolimus has been associated with the risk of presenting secondary posterior reversible encephalopathy syndrome, a clinical neuroradiological entity of a benign nature, characterized by neurotoxic effects and mild symptoms such as a headache, tremor, dysesthesia or pseudo-perceptions, and self-limiting course [31]. Since liver transplant candidates and recipients are at a high risk of psychological distress, the most recent approaches to this topic underline the importance of psycho-social and psychiatric patterns, including the evaluation of the patient’s psychological strengths, limitations, and needs [32,33,34]. After the transplant, personalized psycho-social interventions favor recovery processes, improving the quality of life and adherence to immunosuppressants [32,33].

In the case presented here, there are significant affective and behavioral manifestations that required adjustment by psychoactive drugs. The patient presented obsessive thoughts, delusions, hallucinations, and important intrusiveness that affected his social life. Other symptoms, such as motor disorders and functional deficits, resulted in his admission to hospital. However, the history of prior depressive disorder, multiple pathologies, and pharmacological issues (polypharmacy and drug–drug interactions), and issues such as pharmacokinetics and pharmacodynamics conditioned by the different situations of the patient, may question the hypothesis on etiopathogenic. Despite this, we presume that an immunosuppressive treatment (the use of cyclosporine and everolimus) is linked to the progressive and recurrent behavioral symptomatology. Psychotic, affective, and manic symptoms in different types of organ transplants have been linked to the suppression of cellular immunity and the inhibition of the proliferation of T lymphocyte. We ruled out the posterior reversible encephalopathy syndrome as it usually appears within the first weeks, neuroradiological manifestations in the white matter are absent, and it is usually associated with the use of tacrolimus [35].

Regarding the psychosocial intervention, the transplantation protocol includes aspects such as the suitability of the donor, the recipient, and the social/family environment of both in a broad sense. The organic, pharmacological, cognitive, personal, and emotional aspects are assessed to help limit the impact of the intervention and ensure a therapeutic adherence [36]. In addition, the pandemic-related health context and social distancing measures have led to changes in psychogerontological intervention strategies and programs, e.g., individualized and home interventions have proliferated. In turn, rapid technological advances have allowed for the creation of digital cognitive training and rehabilitation programs. The use of technology-based interventions improves the cognitive and behavioral outcomes of people with a mild cognitive impairment and behavioral disorders [37], providing more immersive and complete user experiences and promoting adaptive responses based on an individual performance [38]. A brief and rapid intervention has been possible through the easy access to computerized cognitive intervention programs that provide real-time feedback to both participants and professionals [39].

Regarding the post-intervention evaluation, although the cognitive performance and subjective memory complaints improved, the exacerbation of behavioral, psychotic, and emotional symptoms seem to have conditioned the performance of executive functions (Table 2 and Table 3) because the pharmacological treatment had to be increased during the intervention.

Regarding the psychopharmacological treatment, the incidence of delusional activity, agitation, and anxiety conditioned the prescription of atypical antipsychotic drugs such as quetiapine and olanzapine. Olanzapine was the main antipsychotic, justified for its proven efficacy (in schizophrenia, mania, and bipolar disorder) and because it is the only second-generation drug that is not metabolized by CYP 450 3A4. However, some associated adverse effects, e.g., hyperprolactinemia, may increase the risk of rejection [40], in addition to the metabolic side effects, increased appetite, and secondary sedation. As for the complementary treatment with quetiapine, the reason for its prescriptions must have been its efficacy in mania and bipolar disorder and wide range of doses; again, its frequent side effects, e.g., orthostatic hypotension, dizziness, and liver disorders, should be considered in cases like the one we report here [41]. Clonazepam is an alternative agent against anxiety reactions, with a rapid effect and manageability, adverse effects, e.g., sedation, ataxia, or effects on the memory or motor performance, should be considered [41].

No SSRIs was prescribed to the study patient, which might seem surprising, given the presence of affective symptoms. The coexistence of other risk factors such as metabolic and cardiac disorders [42], and the potential increase in the concentration of calcineurin inhibitors and its secondary toxicity or the possibility of inducing paradoxical reactions and hypomania, would justify this decision [43].

## 4. Conclusions

The immunosuppressive treatment given to the patient, needed to ensure his survival, is associated with neurotoxicity and neuropsychiatric symptomatology. In our case, the response to an immunosuppressive treatment shows a good adaptation, adequate transplant tolerability, and an organic and analytical clinical stability, but secondary neuropsychiatric symptoms including psychological (depression, anxiety, and delusions) and behavioral (irritability, agitation, and intrusiveness) symptoms. These neuropsychiatric symptoms required a follow-up and multifactorial psychopharmacological treatment in addition to a psychosocial intervention. A possible rejection of the use of psychoactive drugs, including Olanzapine, has not been shown. Potential side effects such as dyslipidemia, hyperglycemia, and weight gain have been monitored in the different consultations and complementary tests carried out without finding deviations of interest. In these cases, a close clinical follow-up and the monitoring of the immunosuppressive drug therapy should be carried out during medium-term patient management.

Neuropsychiatric symptoms have a critical impact on the life and well-being of patients and their family. Thus, the differential diagnosis should be considered with a thorough medical history and treatment analysis as well as the mental status and neurological examinations. Moreover, suspected acute processes deserve CT scanning, but MRI imaging is more sensitive to a subtle structural change. Nonetheless, the radiographic findings could not explain the patient´s symptoms. The management of neuropsychiatric symptoms should always need an assessment of the patient for the medical and environmental causes of their behavior. However, if the problem persists, nonpharmacological interventions should be attempted before using drug therapy. If drug therapy is to be established, we need to bear in mind that it has its merits and pitfalls. One approach to choose psychopharmacology medication could be to identify the target symptom. An alternative method is the one guided by the current state of evidence in combination with the goal of minimizing the adverse effects. There is no “magic pill” for the control of neuropsychiatric symptoms, and due to that, it is vital to continue the efforts to better understand the pathophysiology of the symptoms.

In sum, this case showcases the vital importance of the identification and treatment of neuropsychiatric symptoms since they have a significant impact on the life and well-being of patients and their loved ones.

## Figures and Tables

**Table 1:** Pharmacological treatments received by the patient from 2020 to 2022, including medication adjustments.

Drugs/Year	2 October 2020	5 October 2020	11 January 2021	23 March 2022
Immunosuppressants				
Cyclosporine	150	150	150	50
Everolimus	2	2	2	1.5
Antiarrhythmics				
Bisoprolol	2.5	2.5	2.5	2.5
Amiodarone	100	100	100	-
Diuretic				
Furosemide	20	20	20	40
Treatment for heart failure				
Sacubitril/Valsartan	48/52	48/52	48/52	24/26
Antiplatelet agents				
Acenocumarol	BL	BL	BL	BL
Hypolipidemic				
Atorvastatin	-	-	-	20
Proton-pump inhibitor (PPI)				
Omeprazole	-	-	-	20
Vitamins				
Folic acid	5	5	5	5/week
Pyridoxine hydrochloride	150	150	150	150
Oral antidiabetics				
Empagliflozin	-	-	-	10
Anticonvulsivants				
Lacosamide	300	300	300	300
Clonazepam	4	2	2	3
Neuroleptics				
Quetiapine	100	100	100	100
Olanzapine (orodispersible tablets)	15	15	15	15
Olanzapine	-	-	-	10
Tiapride	600	500	-	-
Anti-potassium drug				
Patiromer	-	-	-	33.6
Laxative				
Plantago ovata	-	-	-	3.5

Doses shown in milligrams; BL: blood levels.

**Table 2:** Assessment of patient’s mental status before and after the cognitive intervention.

	Test	Pre-Intervention Score	Post-Intervention Score
Objective cognitive status	MoCA	20	22
	FAB-E	10	9
Subjective cognitive status	MFE	14	9
Neuro-psychiatric symptoms	NPI-Q	32	43
	MBI-C	19	26
Depressive symptoms	Cornell scale	12	13

**Table 3:** Results of the application of the STIMULUS program.

Session	Exercises	Stimulus Score	Result	Execution Time (ms.)	Right Answers	Mistakes	Omissions	Average Response Time
1	40	24.51	79.30	2054.14	80.45	10.65	10.38	8.42
2	39	28.53	76.95	2349.33	78.44	11.26	14.49	8.56
3	41	28.34	78.56	2172.54	79.05	12.15	11.34	6.05
4	41	29.79	85.71	2287.40	86.34	7.41	8.46	5.04
5	49	28.76	83.16	2145.15	83.39	5.16	12.53	3.87

## Data Availability

The data presented in this study can be requested to the corresponding author. The data are not publicly available due to confidentiality and anonymity considerations.
